# Management of plica polonica in the wake of refractory head lice infestation: a case report

**DOI:** 10.1186/s41182-026-00920-y

**Published:** 2026-02-09

**Authors:** Nirin Seatamanoch, Sakone Sunantaraporn, Padet Siriyasatien, Chanat Kumtornrut, Narisa Brownell

**Affiliations:** 1 Department of Parasitology, Faculty of Medicine, Chulalongkorn University, Bangkok, Thailand; 2 Center of Excellence in Vector Biology and Vector-Borne Diseases, Department of Parasitology, Faculty of Medicine, Chulalongkorn University, Bangkok, Thailand; 3 Division of Dermatology, Department of Medicine, Faculty of Medicine, Chulalongkorn University, and King Chulalongkorn Memorial Hospital, Thai Red Cross Society, Bangkok, Thailand

**Keywords:** Pediculus humanus capitis, Head lice, *Kdr* mutations, Permethrin, Pediculosis

## Abstract

**Background:**

Pediculosis capitis remains widespread globally, and the emergence of resistance genes continues to impede eradication efforts. This case of plica polonica offers insight into the complexity and challenges of pediculosis in endemic settings where resistant lice are present.

**Case presentation:**

We report the case of a 13-year-old girl from a suburban area of Bangkok with long-standing pediculosis that progressed to plica polonica due to prolonged lack of intervention. Molecular analysis of the sampled lice demonstrated a 100% homozygous knockdown resistance (*kdr*) mutation. Several of her classmates also described recurrent infestations despite previous permethrin use, and permethrin exposure in this case showed limited effect, as lice remained viable after the recommended treatment period. The condition resolved after incorporating physical modalities.

**Conclusion:**

Pediculosis in endemic areas is at a high suspicion for resistance. Chemical treatment alone may not lead to a cure, and caretakers should emphasize other treatment forms to achieve a cure. Such an approach may help prevent further mutations due to inbreeding within the headlice population with resistance genes from the unoptimized chemical treatment.

## Background

*Pediculus humanus capitis* is an obligate human ectoparasite feeding on human blood [1]. This report describes a case of plica polonica as a consequence of lack of grooming from pediculosis, complicated by secondary bacterial infection, identified in an endemic region with documented high permethrin resistance. Current evidence remains insufficient to establish a definitive genotype–clinical responsiveness in cases with *kdr*-mediated resistance [2]. Nonetheless, this case highlights an approach that may be applicable in endemic settings.

## Case report

A 13-year-old girl from Nonthaburi, Thailand, presented with a one-month history of scalp pruritus, followed by visible lice infestation, burning sensation, and rash on both ear helices. Due to fear of touching the lice, she had abstained from washing or grooming her hair, which remained tied in a ponytail. She avoided school due to embarrassment. Previous episodes of pediculosis had been successfully treated with Thai traditional medicine, which involves the application of custard apple leaves prepared in rice whisky and via hair shaving. Other classmates had a prior history of head lice infestation and were periodically treated with insecticidal products.

**Fig. 1:**
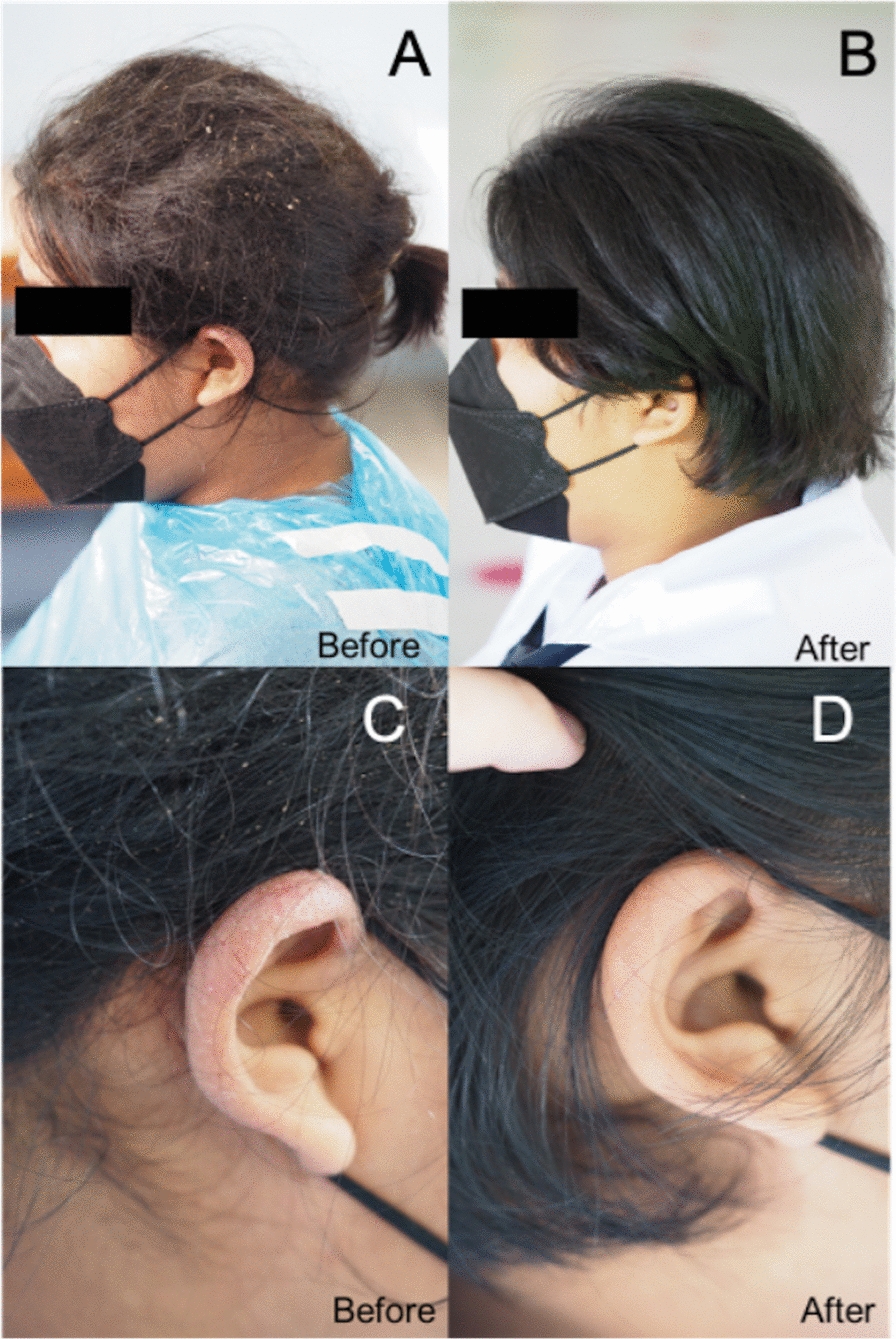
Clinical improvement observed after 2 weeks from the last treatment. Post-treatment, plica polonica and pediculosis capitis completely resolved. Only minimal empty nits were found. The patient regained her self-confidence and returned to class (**A**, **B)**. After pediculosis capitis treatment, eczema at both ear helices cleared out without the use of any other medications (**C**, **D)**

Examination revealed extensive lice infestation, numerous nits, flaky dandruff, and concretions concentrated in matted hair masses. The scalp exhibited widespread erythema accompanied by exudate and signs of secondary bacterial infection. Both ear helices showed eczematous plaques (Fig. 1A, C).

**Fig. 2:**
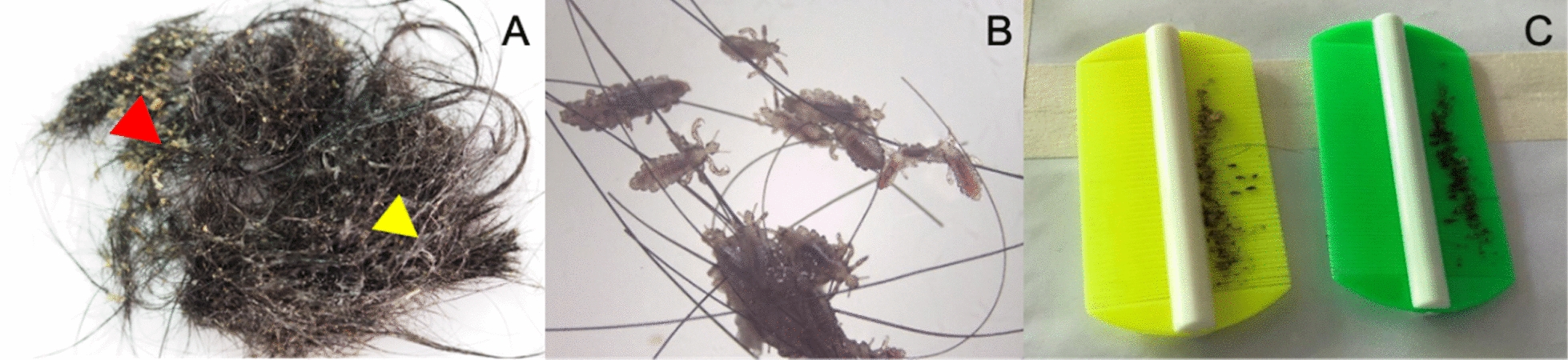
The irreversibly matted hair, similar to a bird’s nest, was observed. Adult head lice, nits, and dandruff were grossly visible. One of the trimmed hair masses displayed honey-colored crusts adhered to the hair (red arrow), and the white liquid material was hair conditioner (yellow arrow) (**A**, **B)**. Head lice, nits, and debris were removed by fine-tooth combs after medical treatment and haircut (**C**)

Treatment included 1% permethrin lotion on days 0 and 7. On day 0, hair conditioner was applied to facilitate detangling, and partial haircutting was performed to address severe matting. Following a 10-min application of permethrin, a considerable number of adult lice persisted despite a thorough application. Fine combing, along with repeated shampooing, was subsequently employed to remove the lice and associated debris (Fig. 2). The mechanical removal using a fine-toothed lice comb at home was carried out daily before the subsequent follow-up visit. By day 7, no live lice were detected. Complete resolution was achieved 2 weeks later, with clearance of eczematous lesions and restoration of regular hair hygiene, allowing the patient to return to school (Fig. 1B, D).

Molecular analysis of ten sampled lice revealed a homozygous knockdown resistance (*kdr*) mutation in all specimens, indicating permethrin resistance.

## Discussion and conclusions

Pediculosis capitis can significantly affect both physical and mental health. This case highlights a 13-year-old girl with pediculosis capitis complicated by plica polonica, a rare condition characterized by the gradual formation of dense, matted hair masses. Plica may result from physical, chemical, or behavioral factors [3]. In this case, prolonged lack of care of scalp hygiene following infestation contributed to disease progression. The ear-helix eczema, likely an id reaction secondary to impetiginization, resolved spontaneously after pediculosis eradication [1].

Considering the patient’s extensive clinical involvement, oral ivermectin may be the preferred treatment option due to the limited penetration of topical agents through matted hair [4] However, the patient desired rapid resolution of plica polonica but expressed concern regarding systemic therapy. Therefore, permethrin lotion and a haircut were selected as the initial treatment. The persistence of live lice immediately after permethrin application, combined with local outbreak history, raised suspicion of resistance, later confirmed molecularly [5, 6]. For our patient, apart from the medication, meticulous wet-combing and partial haircutting contributed to the cure, highlighting the importance of multimodal strategies when managing resistant or complicated cases. Even in comparatively uncomplicated cases, patients who acquire pediculosis in endemic regions may not respond adequately to chemical therapy alone. Reliance on pharmacologic treatment without adjunctive measures risks suboptimal eradication and may further facilitate the emergence and propagation of resistance-associated genes due to interbreeding between head lice with and without resistance genes within the community population [7]. Another common concern in cases such as ours is the risk of reinfestation. Beyond the negative impact on the individual patient, sustained prevalence of head lice within a community may increase the likelihood of resistance gene development and dissemination on a larger scale [7]. Community education and awareness campaigns should therefore be strongly emphasized. However, routine genetic screening may not be the first-line approach for managing complicated cases due to limited clinical relevance, limited access, and cost.

This case report does not suggest that plica polonica associated with pediculosis is directly linked to resistance genes. In our patient, the condition primarily resulted from prolonged lack of care. However, additional cases arising from prolonged pediculosis may occur as a result of treatment failure. It is important to recognize that treatment failures at the community level may also stem from inadequate therapeutic strategies, particularly in addressing potential resistance mechanisms.

## Data Availability

The data that support the findings of this study are available from the corresponding author, NB, CK, upon reasonable request.

## Abbreviation

*Kdr*: Knockdown resistance
